# Time-varying impact of snow depth on tourism in selected regions

**DOI:** 10.1007/s00484-019-01848-1

**Published:** 2019-12-28

**Authors:** Martin Falk, Xiang Lin

**Affiliations:** 1 School of Business, Department of Business and IT, University of South-Eastern Norway (USN) , Bø, Norway; 2 School of international economics and trade, Shanghai Lixin University of Accounting and Finance, Shanghai, China; 3 Södertörn University, Huddinge, Sweden

**Keywords:** Domestic tourism demand, Overnight stays, Winter tourism, Snow depth, Time-varying models

## Abstract

This study uses a time-varying model that provides new evidence on the changing relationship between domestic overnight stays of selected winter sport destinations and natural snow conditions. A Kalman filter method combined with wavelet-based multiresolution analysis (MRA) is employed to investigate the relationships in intervals between 2 and 4 and up to 16–32 months. The model is applied to domestic overnight stays for selected mountain regions in Sweden (Dalarna and Jämtland), Norway (Buskerud, Hedmark, Hordaland and Oppland) and Austria (Salzburg and Tyrol). Results show that the sensitivity of domestic overnight stays on natural snow conditions varies markedly depending on location, time period and frequency band window used in the estimation. The medium-run relation for Tyrol and Salzburg is significantly declining over time, while in Norway and Sweden, the same relationship is generally volatile and not significant at the end of the sample period. In the short run, none of the regions exhibits a link between domestic overnight stays and snow depth fluctuations.

**Electronic supplementary material:**

The online version of this article (10.1007/s00484-019-01848-1) contains supplementary material, which is available to authorized users.

## Introduction

According to SMHI (Sveriges meteorologiska och hydrologiska institut), the maximum snow depth during the winter season between 1991 and 2014 decreased compared to 1961–1990 in all of Sweden except Norrland (Wern 2015). Similar trends can be observed for Western Austria (ZAMG 2017). Many studies have investigated the relationship between fluctuations in snow depth and tourism inflows to winter sport destinations using econometric models (Falk 2010; Töglhofer et al. 2011; Damm et al. 2017). However, the assumption that the parameters of the relationship between climate variability and tourism inflows are stable over time is not realistic, as extreme winter seasons can lead affected ski lift operators and destinations to intensify their adaptation measures, making the tourism sector less vulnerable to climate variability.

The aim of the study is to investigate the relationship between natural snow depth and winter tourism, measured as domestic overnight stays. Particular attention will be paid to whether the relationship changes over time. Wavelet models in combination with the Kalman filter technique are used to analyse the temporal variation of the relationship between overnight stays and snow conditions. The data are based on seasonally adjusted monthly data for the winter season at regional level for selected European regions in Austria, Norway and Sweden for a period of up to 40 years.

The effects of climate change and climate variability on ski tourism and snow-based winter tourism are comparatively well studied (Steiger et al. 2019). The authors show that the impacts can vary greatly across regions and over time. However, most studies do not explicitly examine whether the dependence of the winter sports destination on natural snow conditions has changed over a longer period of time.

The main contribution of this study is an analysis of the time-variable relationship between snow depth and tourism inflows, which at the same time makes it possible to distinguish between short-, medium- and long-term impacts. Although there are several studies that investigate the relationship between snow depth and tourism demand (Falk 2010; Töglhofer et al. 2011; Damm et al. 2017), no study has explicitly examined the stability of the relationships over time using advanced time-varying models such as wavelet methods. The other contribution is that comparable data on domestic overnight stays are used for selected European mountain areas over a longer period of time. The study focuses on eight regions in three European countries. The reason for this is the easy access to consistent time series on snow depths and a dense network of weather stations in winter sports regions.

## Conceptual background and previous literature

Regionalized climate models for the Scandinavian mountains predict a temperature rise of about 5 °C in the winter months (December to February) in the period 2071–2100 compared to the period 1971–2000, based on the high emission scenarios (RCP 8.5) (EURO-CORDEX) (Strandberg et al. 2014; Fig. 17). For the European Alps, the expected temperature rise of 3–4 °C is also above average based on RCP 8.5. Thus, mountain regions are particularly affected by global warming in the winter season (Intergovernmental Panel on Climate Change (IPCC) 2014). The maximum snow depth during the winter season decreased between 1991 and 2014 compared to 1961–1990 in the European Alps (Marty 2008; Klein et al. 2016 for Switzerland; ZAMG 2017 for Austria). The climate scenarios for the Alps in the coming decades predict a reduction in the number of days with snow cover and a greater proportion of days. As temperature increases are expected to continue in this century, stakeholders and affected destinations have expressed concerns about possible negative consequences for winter tourism in the Alps. Therefore, knowledge of historical relationships and their changes over time is important to predict future relationships.

The empirical literature shows that both differences in the climate zone between origin and destination as well as climate variability have significant effects on tourism flows (Goh 2012; Li et al. 2016; Li et al. 2017; Agiomirgianakis et al. 2017; Zhang and Kulendran 2017; see Becken 2013a, Fang et al. 2018 and Hewer and Gough 2018 for surveys). Other studies investigate the tourism effects of extreme weather seasons (Gómez-Martín et al. 2014 for the summer season and both Steiger 2011 and Rutty et al. 2017 for the winter season) and find that extreme seasons have negative impacts on tourism inflows. Some studies find that variability of visitor nights over years is independent of weather conditions (e.g. Becken 2013b).

In general, studies can be distinguished by season (winter or summer), region (lowland or mountains) and frequency (daily, monthly or annual data). With regard to winter tourism, many studies have examined the relationship between demand for downhill skiing and snow conditions and other weather indicators. Studies based on daily data include Hamilton et al. (2007); Shih et al. (2009); Damm et al. (2014); Demiroglu et al. (2015); Malasevska et al. (2017); and Mayer et al. (2018). These studies naturally lead to significant and sometimes large estimates of the impact of natural snow and other weather conditions on skier visits. A main feature of these studies is that they are limited to a few winter seasons (usually between three and nine winter seasons except Shih et al. (2009) which use up to 18 winter seasons). However, when analysing the impact of climate change on winter tourism, a longer period of time is needed to examine whether the relationships change over time. In fact, Gómez-Martín (2005) argues that the data frequency used is crucial for estimating the tourist impact of weather conditions. Over a longer period (e.g. an entire winter season), the weather tends to have little influence on tourism flows. The reason for this could be that tourists are to some extent flexible and can postpone their holiday plans if weather conditions are not suitable for the planned outdoor activities.

Empirical studies that are based on data for an entire winter season over a longer period show a significant relationship between climate variability and winter tourism demand (Falk 2010, 2013; Töglhofer et al. 2011; Falk and Vieru 2019). For instance, Falk (2010) investigates the relationship between overnight stays, relative prices, real income and climate variability using annual variations in weather indicators and dynamic panel data methods. The author shows that demand for winter tourism depends positively on real income and snow depth and negatively on prices. However, the relationship between natural snow depth and the demand for winter tourism is relatively small. Falk (2013) finds that the sensitivity of winter guests to climate fluctuations is more pronounced among domestic guests than among foreign guests, although the absolute degree of sensitivity is quite low. Damm et al. (2017) is the only study that uses data for a large number of regions in Europe. The results show that total overnight stays in 56 of 119 NUTS 3 regions depend significantly on snow depth (at the 5% significance level). However, the period of the estimation sample is too short to test the stability of the parameters over time.^1^

Studies that measure performance by lift transportation or skier visits lead to similar results (Gonseth 2013; Falk 2015; Falk and Hagsten 2016; Falk and Vieru 2017; Falk and Lin 2018b). Studies include, for example, an analysis for the largest French ski lift operator (Compagnie des Alps, CDA) (Falk 2015), and studies for the Swedish ski industry (Falk and Hagsten 2016; Falk and Lin 2018b). Gonseth (2013) use data for Swiss ski resorts and find that the number of skier days depends on snow conditions, with a greater impact on ski resorts with lower snow-making capacity. Falk and Vieru (2017) document that the relationship between natural snow depth and the performance of ski lift companies depends on the location (here latitude), with a greater impact on locations in lower latitudes. Thus, models based on aggregate data for the whole country hide differences between locations. Falk and Hagsten (2017) show that climate change also threatens other snow-covered winter tourism activities such as cross-country skiing.

Overall, few studies investigate the relationship between the variations in snow conditions and overnight stays in winter destinations that use a long period of time, partly due to the lack of consistent time series for snow depth and overnight stays. However, a longer period of 40 years or more is needed to study the effects of climate change. Falk and Lin (2018a) find that the relationship between tourism demand and temperatures in the winter season weakens over time. The data is based on time series of arrivals for the winter season 1960–2015 in South Tyrol (Italy). Falk and Lin (2018b) find similar results for the output of the Swedish ski lift companies. However, these studies cannot distinguish between short-, medium- and long-run effects and refer to single region (Swedish mountains and South Tyrol), and standard tests of the stability of parameters are used (recursive coefficients method, Chow test). For the ski industry in the USA and Austria, Dawson et al. (2009) and Steiger (2011) suggest that negative demand effects due to lack of snow or exceptionally high winter temperatures decrease over time. This indicates that the ski industry is becoming increasingly independent of climate fluctuations.

Another strand of the literature investigates possible reactions of winter tourists with respect to natural snow conditions using surveys and experiments (Unbehaun et al. 2008; Rutty et al. 2015; Pons et al. 2017). These studies confirm that preferences for skiing activities are influenced by the lack of snow (Unbehaun et al. 2008; Rutty et al. 2015). However, these studies can be criticized because they are carried out at a certain point in time and the response behaviour is subjective and may be influenced by snow conditions during the study period or before. In fact, Trawöger (2014) summarize the findings of several surveys on the impact of climate change on winter tourism and emphasized that respondents are influenced in their responses by past exceptional weather conditions either in summer or winter.

In summary, previous studies show that, despite strong investment in snow-making equipment over the last 25 years, demand for skiing or winter tourism in general still depends on fluctuations in snow depth in the ski area. However, the size of the relationship between snow depth and overnight stays seems to be rather small and declining over time.

Nevertheless, the assumption that the parameters of the relationship between climate variability and tourism inflows are stable over time is often unrealistic. Extreme winter seasons can induce affected businesses or destinations to intensify their adaptation measures, making the winter tourism industry less vulnerable to climate variability. To mitigate the effects of climate change, winter sports destinations have invested heavily in snow-making equipment and other adaptation measures (snow management and storage, etc.) since the early 1990s (Steiger and Mayer 2008).^2^ Winter seasons with extreme weather conditions can lead to an intensification of adaptation measures. Therefore, the parameters cannot be considered stable over time. Visitors can also get used to the winter season with poor natural snow conditions. They can still go on winter holidays despite poor snow conditions, but they might focus on activities that are not dependent on natural snow activities, such as hiking.

In this case, the impact of climate variability on tourism demand should be small. The use of time-varying models can also account for possible changes in guest behaviour that can occur abruptly when certain thresholds are reached, rather than as a continuous, slow change (Gössling and Hall 2006). This also suggests a time-varying non-linear relationship.

Overall, this means that the relationship between snow depth and overnight stay will become weaker over time. Adaptation measures, however, make little sense in areas specializing in cross-country skiing, ski mountaineering activities and snowmobiling, as they are too time-consuming and too expensive. This suggests that the connection varies from winter sports destination to winter sports destination, with regions focusing on cross-country skiing and snowmobiling, such as the Norwegian and Swedish regions, being more vulnerable to lack of snow. The main hypothesis is that technological progress and adaptation of affected businesses, as well as changes in consumer behaviour, can weaken relationships over time, but the effect varies from region to region. In addition, global warming has accelerated recently, which could lead to greater impacts of climate variability in recent years.

This study uses aggregated data at the regional level. Regional data have advantages and disadvantages over destination-specific data such as data for villages or ski resorts. The higher aggregation level should at least partially take into account for possible substitution effects between different ski resorts within a region. In the European Alps, visitors are more likely to move to higher elevations in a given region in winter seasons with a lack of snow. In the Scandinavian mountains, winter sport tourists can only switch to higher areas to a limited extent but could switch to higher latitudes (e.g. northern Dalarna instead of the southern part). Studies based on data for Australia and Austria show that higher elevation resorts experienced a considerably lower decline in winter seasons with poor snow conditions (Pickering 2011; Steiger 2011). This could be partly related to a possible substitution effect from low elevation ski resorts to high-mountain ski resorts. The possible substitution effects could be more pronounced for local and domestic visitors than for foreign visitors. Domestic winter sport tourists are to a certain extent more flexible in terms of travel time, length of stay and destination selection under different snow conditions than foreign tourists. On the basis of panel data for Austrian villages for the winter seasons 1986–1987 to 2007–2008, Falk (2013) provides empirical evidence that domestic overnight stays react more strongly to changes in snow depth than foreign overnight stays. It may also be the case that in both low-lying and high-mountain areas, the number of overnight stays will decrease in snow poor winter seasons, as the interest in skiing is generally lower in winter months with little snow. In order to take account of the possible substitution effect, data for the whole region are used.

## Empirical model

In order to investigate the relationship between tourism demand and snow depth, a tourism demand model is specified. The study focuses on domestic tourists for several reasons. Taylor and Ortiz (2009) suggest that domestic tourists are more influenced by climatic factors as the planning period for domestic holidays is shorter.

According to the theory of tourism demand, overnight stays depend positively on real income and negatively on prices (Song and Witt 2012). In addition to real income and prices, natural snow conditions are generally regarded as an important factor in winter tourism demand (Falk 2010; Töglhofer et al. 2011; Damm et al. 2017). The static tourism demand model can be specified as follows:

1 $$ {lnONS}_t=C+{\alpha}_1\left(t/T\right){lnY}_t+{\alpha}_2\left(t/T\right){lnCPI}_t+{\alpha}_3\left(t/T\right){lnSD}_t+{\varepsilon}_t $$

where *t* denotes the month t, *ln*() is the natural logarithm, *ONS* denotes domestic overnight stays in accommodations and *Y* is a proxy of domestic real income and is measured as the retail sales volume index. *CPI* denotes the consumer price index and *SD* is snow depth. All series are seasonally adjusted. The parameters *α*_1_(*t*/*T*), *α*_2_(*t*/*T*) and *α*_3_(*t*/*T*) are allowed to vary over time. The equation contains the error term *ε*_*t*_.

The question arises as to how the time-varying model is estimated. One estimation method is the time-varying cointegration (TVC) method (Bierens and Martins 2010; Falk and Lin 2018c). However, the method is difficult to use when there is a mixture of stationary and non-stationary variables. The TVC method also requires fairly long time series. The idea in this paper is to implement the wavelet-based multiresolution analysis (MRA) to decompose the time series into various detailed components in the corresponding frequency bands and a smooth component. Then the state space model, a set of first-order difference equations of state variables that models dynamic feature of coefficients in our case, is used to estimate time-varying coefficients of these detail components. These coefficients reflect the relations in corresponding frequency bands. The wavelet transformations have widely been used in signal processing. Its applications in economics and finance are recent (see the review of Crowley 2007). Among others, the applications in tourism can be found in Raza et al. (2017) and Sharif et al. (2017).

The wavelet transformation can be regarded as a short-time Fourier transformation that maps the original series to a set of coefficients of the sinusoidal functions. Thus, the series is decomposed into various frequency bands.

This provides advantages of using wavelet transformations over Fourier transformations. One is that wavelet transformations offer information on both time and frequency domains. Secondly, the wavelet transformations can deal with non-stationary series, while the Fourier can only deal with stationary ones.

In this paper, we adopt the widely used Daubechies wavelets which are orthogonal. Generally, Daubechies wavelets maximize the number of vanished moments of the series. The Daubechies wavelets belong to a big family containing various filters. We employ Daubechies least asymmetric scalar filter and wavelet filter of width 8 (LA(8)) (Daubechies 1992). As summarized in Percival and Walden (2006 p. 112), “the least asymmetric filter is the one whose phase function has the smallest maximum deviation infrequency from the best fitting linear phase function”. The width 8 is the lowest in this class. And the LA(8) is suitable for economic studies.

In more detail, the scalar filter is a low-pass filter and the wavelet filter is a high-pass filter. Based on these filters, the wavelet transform can decompose the original time series into smooth parts *S*_*J*_ and detailed parts *D*_*J*_, respectively, at scale *J*. This generates a multiresolution analysis (MRA). According to Mallat’s discrete wavelet transform (DWT) pyramid algorithm, a time series *x*(*t*) can be expressed as MRA:

2 $$ x(t)={S}_J(t)+{D}_{J-1}(t)+\cdots +{D}_1(t) $$

where *S*_*J*_(*t*) is a smooth component at scale *J* and captures cycles and periodicities larger than 2^*j* + 1^; *D*_*j*_(*t*) for *j* = 1,···,*J*, represents detail (periodic and cycle) components of *x*(*t*) at scale *J* or at the frequency band; and 1/2^*j* − 1^ ≤ *f* ≤ 1/2^*j*^. In other words, we can first apply the Daubechies least asymmetric scalar filter and wavelet filter to obtain *S*_1_ and *D*_1_. And then we continue to decompose *S*_1_ into *S*_2_ and *D*_2_ and so on. This paper adopts *J* = 5 for concentrating on the short and medium run. In the case of monthly data, tourism demand is studied at frequency bands of 2(=2^1^) to 4(=2^2^) months, 4(=2^2^) to 8(=2^3^) months, 8(=2^3^) to 16(=2^4^) months and 16(=2^4^) to 32(=2^5^) months. Since we have 5 months (December to April) for one winter season (year), the frequency bands correspond to less than one season, one season, two to three seasons and four to six seasons. Thus, this allows us to distinguish between short run (2 to 4 months) and medium run (4 to 8 months, 8 to 16 months and 16 to 32 months).

In principle, the DWT can deal with seasonal non-adjusted time series (Serroukh et al. 2000). However, this paper intends to use seasonally adjusted data. The reason is that seasonality is treated as noise. Thus, the components at the shortest scale would be disregarded. In our case, we might lose the information concerning the short-term reactions at frequency band 2 to 4 months. The second reason is that the consumption expenditures are already seasonally adjusted. For the consistency of data used here, we carry out seasonal adjustments for the logarithms of overnight stays, CPI and snow depths. We apply the STL procedure developed in Cleveland et al. (1990).

In this study, the determinants of domestic tourism demand are estimated at different scales. After we obtain *D*_*J*_s for logarithms of domestic overnight stays, expenditures, CPI and snow depth, we estimate (1) based on these *D*_*J*_s for *J* = 1, 2, 3, 4 and 5, respectively, by applying Kalman filters aimed at the time-varying coefficients. The results of these coefficients will be reported in graphs.

In addition, as an alternative of the DWT, which is sensitive to the value at initial time point, we also adopt maximal overlap discrete wavelet transform (MODWT), which can handle any sample size and is asymptotically efficient in estimating wavelet variance. Therefore, we carry out the procedure described above to estimate (1) based on maximal overlap of least asymmetric filters with width of 8 MOLA(8). This exercise can be regarded as robust checks for the results obtained based on LA(8).

## Data

The dependent variable is nights spent in accommodation establishments of domestic residents and originates from the National Statistical Offices (Statistics Austria, Norway and Sweden). For Austria, Norway and Sweden, monthly data are available at regional levels from the beginning or end of the 1970s. Overnight stays include hotels and similar establishments, private accommodation and apartments. Specifically, we use domestic overnight stays for two mountain regions in SE (Dalarna and Jämtland), four regions in Norway (Buskerud, Hedmark, Hordaland and Oppland) and two federal states in Austria (Salzburg and Tyrol). Since income data is not available on a monthly basis, we use the domestic retail trade volume as a proxy. Both the retail trade volume and the consumer prices indices at the aggregate level are downloaded from OECD STATS (http://stats.oecd.org/).

**Fig. 1:**
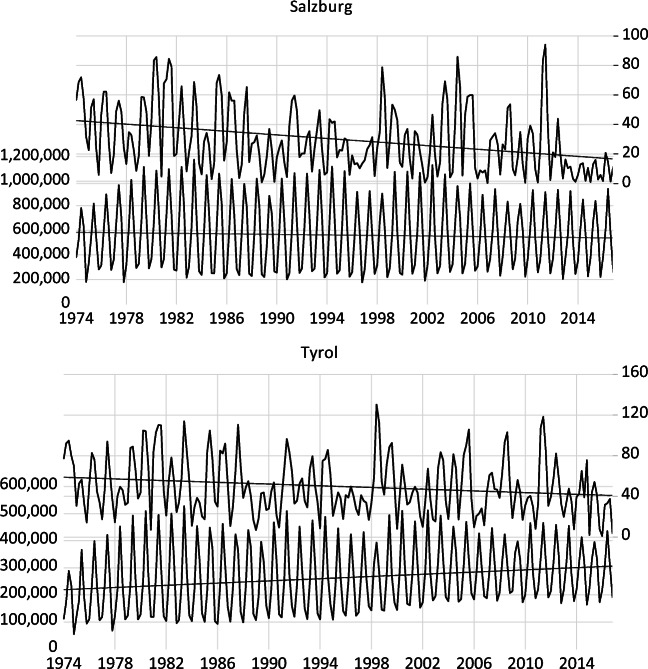
Evolution of domestic overnight stays and snow depth in selected regions in Austria. Note: Average snow depth is measured in centimetres (right axis) and domestic overnight stay is on the left axis. Source: Statistics Austria and Hydrological Service institute

**Fig. 2:**
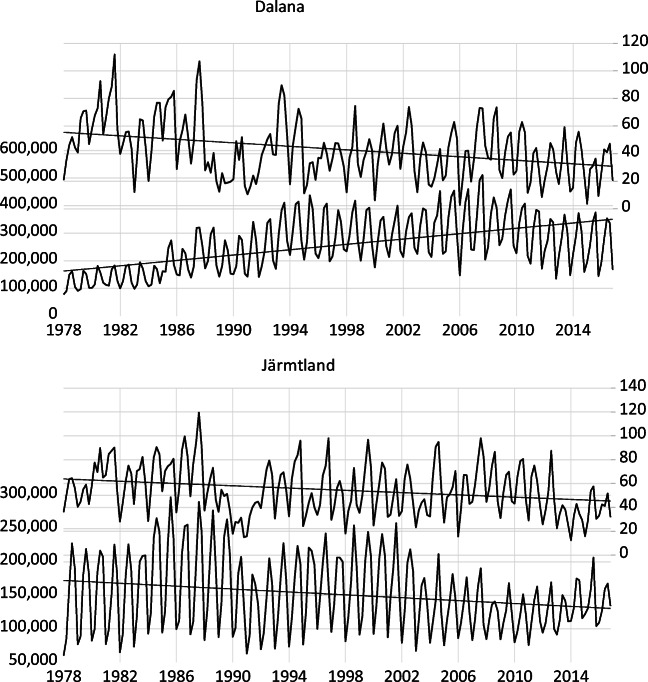
Evolution of domestic overnight stays and snow depth in selected regions in Sweden. Note: Average snow depth is measured in centimetres (right axis) and domestic overnight stay is on the left axis. Source: Statistics Sweden and SMI

**Fig. 3:**
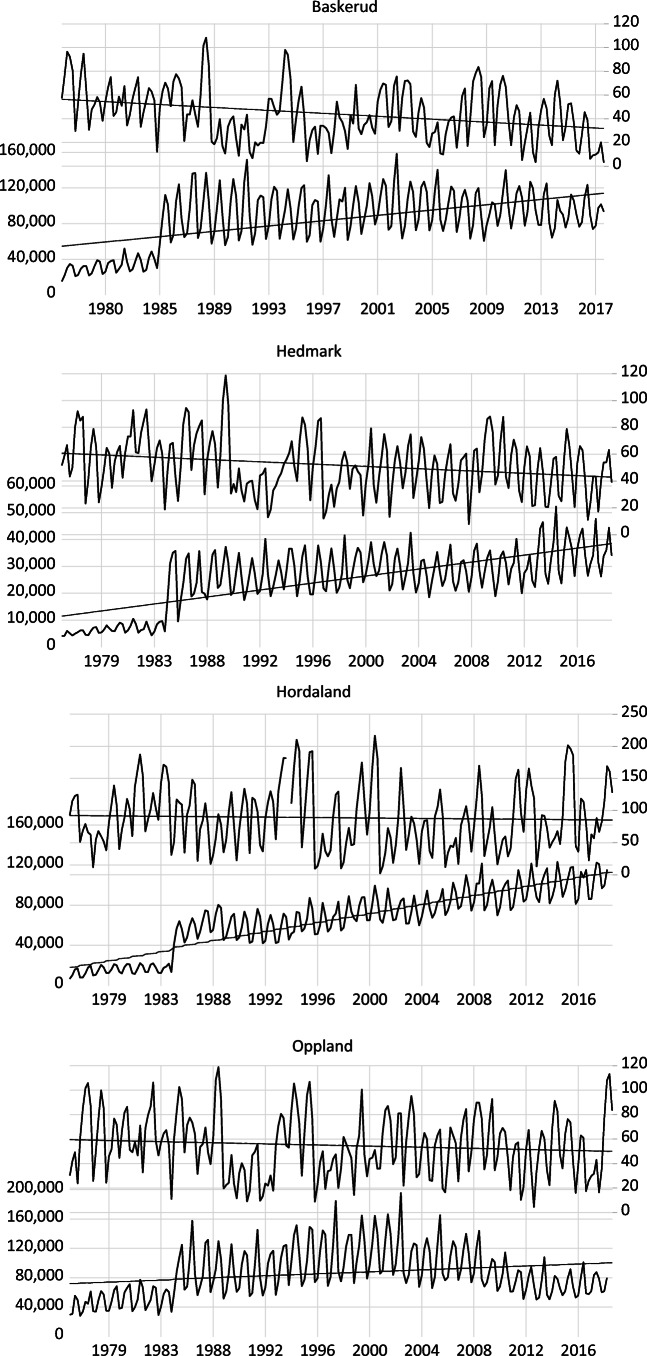
Evolution of domestic overnight stays and snow depth in selected regions in Norway. Note: Average snow depth is measured in centimetres (right axis) and domestic overnight stay is on the left axis. Source: Statistics Norway and NMI

Daily snow depth figures from different weather stations are measured in centimetres and originate from the Norwegian Meteorological Institute, SMI and the Austrian Hydrological Service Institute. There are about 300 weather stations each for Sweden and Austria and about 900 in Norway. Table 1 shows a list of the weather stations used to calculate average snow depth. The selection criteria are the proximity to ski areas or cross-country trails and the altitude. Weather stations in the lowlands are now taken into account. The number of weather stations for each region ranges between 4 and 12. Figures 1 to 3 show the development of domestic overnight stays and the average (non-seasonally adjusted) snow depth in selected European regions. In addition, a linear trend is included. The average snow depth decreases slightly over the period, with the largest decrease in Dalarna, the southernmost region in the Swedish mountain area. In the Austrian federal state, Salzburg, the number of domestic overnight stays is between 200,000 and 1.2 million depending on the month.

**Table 1:** Descriptive statistics

	Mean	Median	Std. dev.
CPI_Austria	75.65	77.79	22.39
CPI_Norway	66.14	68.80	24.11
CPI_Sweden	78.14	85.05	23.08
RVI_Austria	84.84	87.86	13.02
RVI_Norway	72.00	68.27	23.31
RVI_Sweden	71.04	58.98	23.26
ONS_Buskerud	83,148	85,768	32,823
ONS_Dalarna	256,489	243,232	100,770
ONS_Hedmark	25,123	27,064	10,884
ONS_Hordaland	65,771	70,033	30,807
ONS_Jämtland	151,504	141,949	55,619
ONS_Oppland	86,310	79,626	34,029
ONS_Salzburg	560,867	589,509	274,831
ONS_Tyrol	262,787	250,397	119,941
SNOWDEPTH_Buskerud	44.36	42.40	22.77
SNOWDEPTH_Dalarna	43.15	41.57	21.06
SNOWDEPTH_Hedmark	51.82	51.40	21.41
SNOWDEPTH_Hordaland	88.79	84.83	49.32
SNOWDEPTH_Jämtland	54.60	52.24	21.20
SNOWDEPTH_Oppland	54.74	53.25	25.48
SNOWDEPTH_Salzburg	29.68	23.86	22.30
SNOWDEPTH_Tyrol	49.87	43.79	27.84

Notes: ONS denotes domestic overnight stays, CPI is the consumer price index (2010 = 100), and RVI is the retail volume index (2010 = 100). SNOWDEPTH is average snow depth. Source: see text

**Table 2:** Overview of the selected weather stations

Austria	Austria	Sweden	Sweden
Tyrol	Salzburg	Dalarna	Jämtland
Berwang (1295 m)	Boeckstein/Gastein (1092 m)	Idre (455 m)	Höglekardalen (592 m)
Galtür (1583 m)	Bucheben (1034 m)	Närnas (460 m)	Mörsli (400 m)
Gerlos (1260 m)	Filzmoos (1060 m)	Särna (425 m)	Myskelåsen (770 m)
Inneralpbach (1040 m)	Flachau (1000 m)	Sälen (360 m)	Storlien (583 m)
Jochberg (1000 m)	Pass Thurn (1200 m)	Storbron (540 m)	
Ladis-Neuegg (1450 m)			
Lanersbach (1250 m)			
Obergurgl (1939 m)			
See (1063 m)			
Spiss (1628 m)			
St. Anton (1280 m)			
Zürs (1717 m)			
Norway	Norway	Norway	Norway
Buskerud	Hedmark	Hordaland	Oppland
ÅL III (720 m)	Atnsjøen (749 m)	Eksingedal (450 m)	Beito (754 m)
Gol-Stake (542 m)	Blanktjernmoen I Kvikne (700 m)	Fet I Eidfjord (735 m)	Espedalen (752 m)
Geilo (841 m)	Heggeriset-Nordstrand (481 m)	E134 Midtlã (1079 m),	Hovdgrenda (666 m)
Hemsedal (608 m)	Linnes (564 m)	Ger Gullbrå (579 m)	Søre Brekkom (770 m)
Tundhovd (870 m)	Valdalen (794 m)	Jordalen-Nåsen (614 m)	

Source: see text

As expected, Tyrol has a lower number of domestic overnight stays ranging between 100,000 and 500,000 depending on the month considered. The lower number of domestic overnight stays in Tyrol than Salzburg is related to the larger distance to the main population centres in Eastern Austria. In Dalarna and Jämtland, the number of monthly domestic overnight stays ranges between 100,000 and 500,000 and between 50,000 and 300,000, respectively. The Norwegian regions show the lowest number of domestic overnight stays ranging between 40,000 and 200,000. Also, there is a break in the definition of overnight stays in 1983 (for four regions in consideration, the observations before 1983 were below the trends, and a sudden jump occurred in 1983 due to a change in the definition of overnight stays).

## Empirical results

**Fig. 4:**
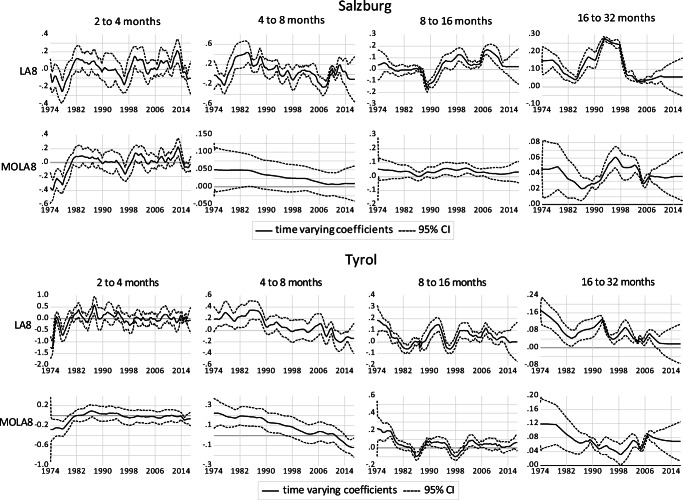
Time-varying relationship between domestic overnight stays and snow depth for Salzburg and Tyrol region

**Fig. 5:**
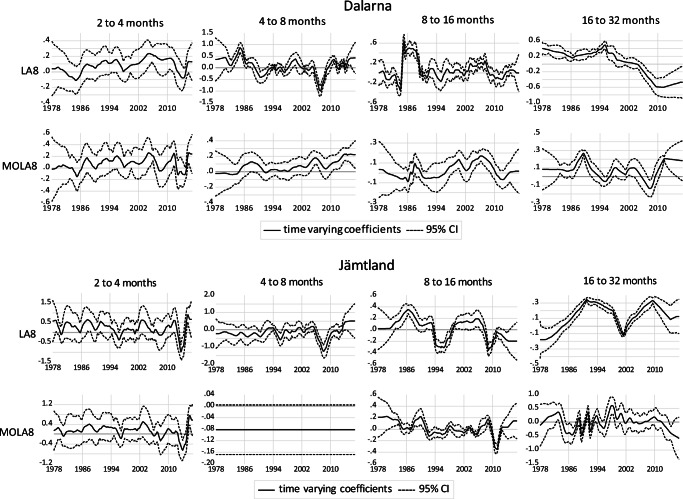
Time-varying relationship between domestic overnight stays and snow depth for Dalarna and Jämtland region

**Fig. 6:**
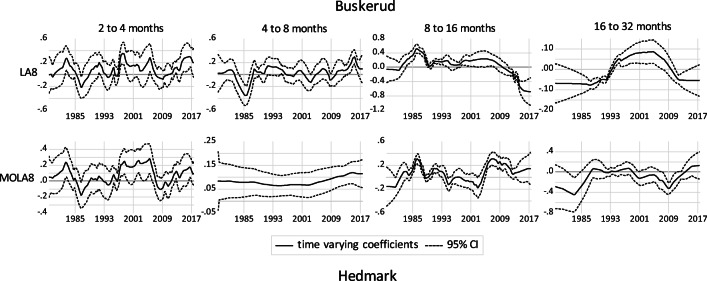

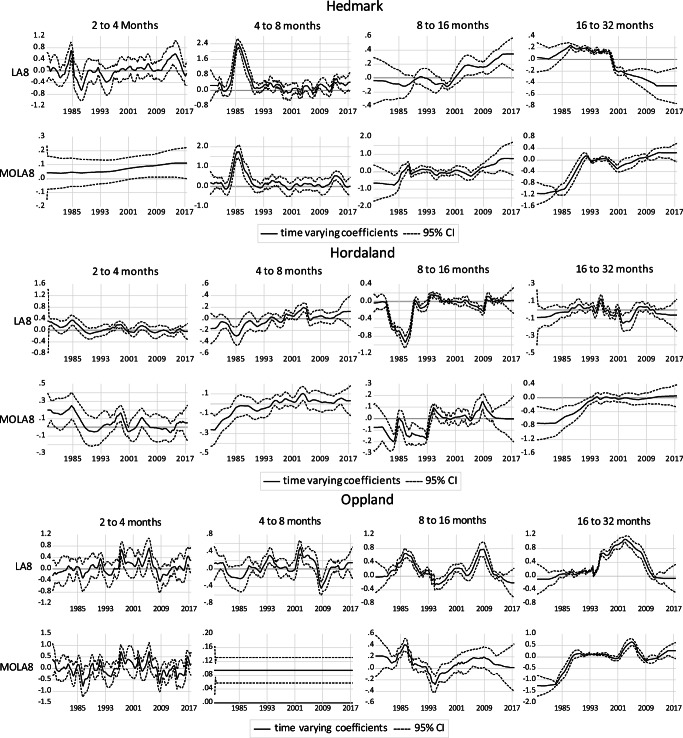
Time-varying relationship between domestic overnight stays and snow depth for Buskerud, Hedmark, Hordaland and Oppland regions

Figures 4 to 6 show the results of the Kalman filter approach combined with wavelet-based MRA for different frequency bands. The graphs show the elasticity of domestic overnight stays with respect to snow depth at four different frequency bands and the corresponding 95% confidence interval. The results are presented for two different transformation methods (LA(8) in the upper panels and MOLA(8) transformations in the lower panels). Results for the two other time-varying parameters (CPI and retail sales volume index) are available upon request. In general, the results show that the dependency of domestic overnight stays on natural snow depths varies widely depending on location, frequency bands and time period. The importance of snow depths for domestic overnight stays is highest in the lowest frequency bands (16 to 32 months). At the short-term level, there is no significant correlation between snow depth and domestic overnight stays, as shown by the lower 95% confidence level, which is below zero in most of the cases.

For Tyrol, the graph shows that the relationship between domestic overnight stays and snow depth decreases over time (upper panel with LA(8) transformations). For instance, the short-run elasticity is about 0.08 on average but no longer at the 5% level from 1996 onwards. Similarly, the medium run of 16 to 32 months shows that snow depth elasticity is falling over time from 0.16 mid-1970s to barely above zero from 2008 onwards. The robustness checks with MOLA(8) transforms shown in the lower panel confirm this in the frequency band of 16 to 32 months. For Salzburg province, no significant relationship between domestic overnight stays and snow depth can be observed in the 4 to 8 months band. In the longer runs, there are clear trends that relationships become insignificant (from 2008 for the lowest frequency in our consideration), similar to Tyrol.

For the Swedish provinces, there is also a tendency that the medium-run elasticities become insignificant in the recent years. This can be observed for Dalarna from 2000 onwards and for Jämtland in recent years (both based on LA(8) and 16 to 32 months frequency bands). In contrast, there are significant positive relationships in the 1980s with elasticities ranging between 0.1 and 0.3.

For the four Norwegian provinces, the medium-run relationships are also not very stable over time. For the regions Oppland and Buskerud, sensitivities in the lowest frequency band (16 to 32 months based on LA(8)) are about to become insignificant in the recent years. For Hordaland, there is no significant positive correlation between domestic overnight stays and snow depths, while for Hedmark the snow depth elasticity decreases over the sample period and becomes significantly negative at the end of the sample period (based on LA(8) and 16 to 32 months frequency band). The results based on MOLA(8) show such tendency exists in all regions. The short-run effects are in general insignificant with exceptions in a few years. Here we also observe stronger relationships over time in some of frequency bands.

The finding that towards the end of our sample the longer-term relationships between snow depth and overnight stays become insignificant is consistent with previous studies (see Töglhofer et al. 2011 and Steiger et al. 2019 for a review). Falk and Lin (2018a) also show that there is a long-run decline in dependency of snow depths in the long-run perspective. However, the study of Falk and Lin (2018a), just like common cointegration studies, does not distinguish between different frequency band windows. A novel contribution of this paper is the explicit focus on various frequency bands for the relationships.

Several robustness tests are performed. Firstly, the possible effect of early Easter is considered by including two dummy variables for the months of March and April when Easter Sunday is in March. However, unreported results show that the dummy variables are not significant. Secondly, for the Austrian regions, data allows estimations including an average of snow depths for valley and summit stations (e.g. Fleissscharte/Sonnblick). Unreported results show that the results are not sensitive to the inclusion of high elevation weather stations.

## Conclusions

This paper provides new empirical insights into the temporal relationship between domestic overnight stays and snow depth. To investigate the temporal variation of the relationship, the Kalman filter method is applied to wavelet-transformed data. The approach is flexible and allows the distinction between short-term and medium-term effects. The results show that the relationship between snow depth and domestic overnight stays varies according to region, time interval (frequency bands) and time period. The main result is that the longer-run relationships between snow depth and domestic overnight stays tend to decline over time and become insignificant in recent years. The declining sensitivities can be particularly observed for Tyrol and Salzburg, while for mountain regions in Sweden and Norway, the relationships are more volatile. This might be due to massive investments in snow-making facilities starting in the 1990s and might be also related to a change in consumer behaviour. An important result of the study is that in the short and medium run, the elasticities of domestic overnight stays on natural snow condition are far from stable. This is clearly related to the method that examines short- and medium-term relations as opposed to long-term cointegration models, which could take 20 years to reach a long-term equilibrium.

Some limitations of this work should be mentioned. Firstly, the results only apply to domestic overnight stays. In some regions, domestic nights account for only a small part of total nights. Therefore, future work should investigate the relationship between international tourism demand and snow depth. Secondly, the results are carried out for eight winter sports areas in Europe and therefore cannot be generalized. Future work should try to include the French, Swiss and Italian Alps as well as winter sport destinations in America and Asia.

## Electronic supplementary material

ESM 1 (XLSX 32 kb).

ESM 2 (XLSX 63 kb).

## Footnotes

1 NUTS means Nomenclature of Territorial Units for Statistics in Europe.

2 Source: annual report of ski lift operators, Skistar
